# A generic approach to estimate airborne concentrations of substances released by indoor spray processes using a deterministic 2-box model

**DOI:** 10.3389/fpubh.2024.1329096

**Published:** 2024-02-09

**Authors:** Stefan Hahn, Katharina Schwarz, Norman Nowak, Janine Schwarz, Jessica Meyer, Wolfgang Koch

**Affiliations:** ^1^Fraunhofer Institute for Toxicology and Experimental Medicine (ITEM), Hannover, Germany; ^2^Unit 4.I.4 Exposure Assessment, Exposure Science, Division 4 Hazardous Substances and Biological Agents, Federal Institute for Occupational Safety and Health (BAuA), Dortmund, Germany

**Keywords:** aerosol, occupational exposure, spraying application, modeling, screening

## Abstract

Sprays are used both in workplace and consumer settings. Although spraying has advantages, such as uniform distribution of substances on surfaces in a highly efficient manner, it is often associated with a high inhalation burden. For an adequate risk assessment, this exposure has to be reliably quantified. Exposure models of varying complexity are available, which are applicable to spray applications. However, a need for improvement has been identified. In this contribution, a simple 2-box approach is suggested for the assessment of the time-weighted averaged exposure concentration (TWA) using a minimum of input data. At the moment, the model is restricted to binary spray liquids composed of a non-volatile fraction and volatile solvents. The model output can be refined by introducing correction factors based on the classification and categorization of two key parameters, the droplet size class and the vapor pressure class of the solvent, or by using a data set of experimentally determined airborne release fractions related to the used spray equipment. A comparison of model results with measured data collected at real workplaces showed that this simple model based on readily available input parameters is very useful for screening purposes. The generic 2-box spray model without refinement overestimates the measurements of the considered scenarios in approximately 50% of the cases by more than a factor of 100. The generic 2-box model performs better for room spraying than for surface spraying, as the airborne fraction in the latter case is clearly overestimated. This conservatism of the prediction was significantly reduced when correction factors or experimentally determined airborne release fractions were used in addition to the generic input parameters. The resulting predictions still overestimate the exposure (ratio tool estimate to measured TWA > 10) or they are accurate (ratio 0.5–10). If the available information on boundary conditions (application type, equipment) does not justify the usage of airborne release fraction, room spraying should be used resulting in the highest exposure estimate. The model scope may be extended to (semi)volatile substances. However, acceptance may be compromised by the limited availability of measured data for this group of substances and thus may have limited potency to evaluate the model prediction.

## 1 Introduction

For manufacturing and marketing chemicals or biocidal products in the European Union, enterprises must fulfill legal requirements. According to European chemicals legislation, a risk assessment is usually necessary, which also includes the assessment of occupational exposure that can be model-based if relevant monitoring data are not available. This model-based exposure assessment often follows a tiered approach, where it is expected that the degree of conservatism for the prediction decreases with increasing levels of detail and accuracy of the prediction. This comprises a series of models with increasing complexity and degree of detail. This means that the models should span a range from simple generic models, which need basic and easily obtained input parameters, to sophisticated (e.g., deterministic or probabilistic) models for which comprehensive information on the processes is required. Depending on the availability of input parameters, the suitable tier level can be selected for the exposure assessment. Due to these different demands and needs, there is a large number of models available for the exposure assessment (1). A review of available models and the status and further needs for modeling spraying activities are given in Hahn, Meyer (2). Spray applications are activities used to atomize liquids into droplets for dispersion of, e.g., pesticides, biocides, and paints (3). Thereby, spraying has several advantages such as uniform distribution of substances on surfaces in a highly efficient manner. However, non-volatile substances will become airborne as aerosols and thus inhalable by these activities. Moreover, the surface area of the products will increase so that volatile substances can more easily evaporate, resulting in potentially higher air concentrations. Tasks such as spraying solvents or pesticides can produce very high exposure levels (3), which are linked to several chronic health impacts such as cancer, neurotoxic effects, or reproductive toxicity. Respiratory effects such as temporary irritation and asthma during spray cleaning and by disinfection products have been discussed by Clausen, Frederiksen (4). The authors found that especially corrosive chemicals are chemicals of concern regarding respiratory effects (e.g., asthma). Furthermore, they concluded that the assessed epidemiological studies provide some evidence of increased asthma risk or worsening of asthma symptoms while using spray cleaning products in a professional or private context. Overall, occupational exposures continue to cause an important health burden worldwide, justifying the need for ongoing prevention and control initiatives (5). Spraying activities are often associated with high inhalation burden, and spray products require additional considerations to assess potential inhalation exposure.

The exposure assessment by modeling of chemicals applied by spray processes is challenging because of the high number of parameters and the variance of their values having influence on the airborne concentration. Especially the higher tier models require full details of the spraying process and the dispersion mechanisms, which are often not available. For this reason, simple model approaches and the improvement of existing spraying models are a valuable addition (6, 7).

The inhalation exposure during spraying is determined by the rate at which the spray is released into the air, the dispersion and the maturation of the released droplets by deposition (mainly settling on horizontal surfaces), and their evaporation. There are two modes of spray application: surface spraying and room spraying. While for room spraying, the entire mass released by the spraying system becomes airborne, only part of it – the overspray or airborne fraction – is available for airborne transport and exposure during surface spraying. The relevance of the exposure-determining mechanisms depends on the spray technology (such as airless versus air assisted, propellant sprays) and the associated parameters such as spray nozzle parameters, spray angle, distance to wall, and droplet size distribution. Further parameters related to the formulation are its chemical composition, the mass fraction, and partial vapor pressures of the relevant compounds (e.g., active substance or pigments and solvents).

In this article, we present a simple tier 1 approach for the assessment of the time averaged exposure concentration using only a minimum of input information and discuss possible refinements based on the classification and categorization of two key parameters: the droplet spectrum and the solvent vapor pressure. The model results are compared with measurements carried out at real workplaces. The degree of conservatism is assessed and discussed. Currently the model is restricted to binary spray liquids composed of a non-volatile fraction and volatile solvents.

## 2 Materials and methods

### 2.1 Modeling approaches

There are numerous approaches available for indoor occupational exposure modeling (1). Only very few of them focus on spray processes (2). A common way to assess indoor exposure concentration by deterministic modeling is to balance the mass flows of sources and sinks inside a closed system.

#### 2.1.1 Generic 2-box spray model

On the lowest level a mass balance model requires knowledge of only a few generic parameters: the source strength of the spray process and the removal rate by air exchange together with the room volume, and spraying and post-spraying duration. Further mechanisms which also determine the air concentration, such as spray maturation by droplet evaporation and mass losses due to droplet settling onto the floor and other surfaces for example, are neglected in this modeling approach.

**Figure 1:**
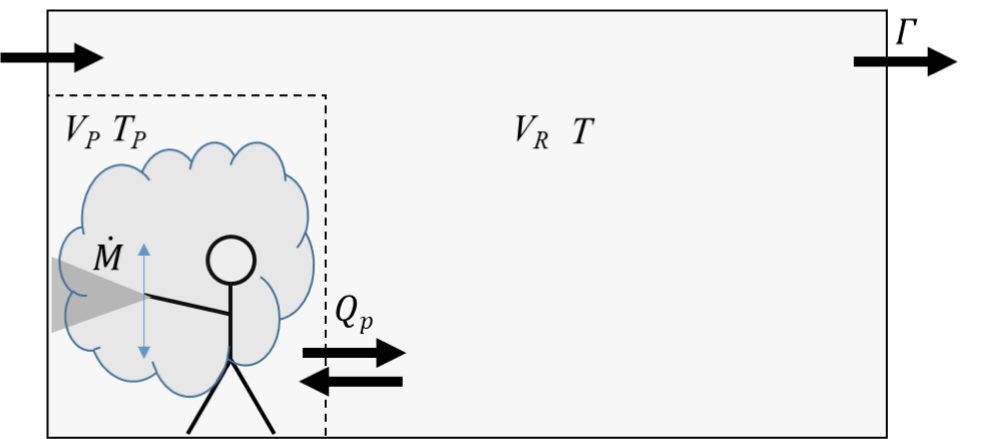
Schematics of the proposed 2-box approach. Assumptions: the personal volume (V_P_) is small compared to the room volume (V_R_), and the residence time (T_p_) of the spray inside the personal volume is small compared to the exposure time T (spraying and post spraying). Q_p_ = 100 m^3^/min, which, for example, corresponds to T_p_ of 0.1 min for V_P_ of 10 m^3^.

The mass balance model suggested here as a tier 1 screening model is based on a well mixed 2-box approach as shown in Figure 1, characterized by a personal volume, $V_{p}$ , and a room volume, $V_{r}$ . We consider a single spraying event of duration, T, composed of a spraying period, $T_{s}$ , and a post spraying time, $T-T_{s}$ . The spray liquid is usually a system composed of N (non-volatile) substances with mass fraction, $\phi_{i}\text{,}$ (total mass fraction, $\phi =\overset{N}{\underset{1}{\sum }}\phi_{i}\ll 1$ ), and solvents with the complementary mass faction, 1−ϕ . The room air is constantly exchanged with exchange rate, Γ . The spray is released at a constant mass flow rate, $\dot{M}\text{.}$

In the 2-box approach, the near field is defined by a personal volume, $V_{p}$ , which is fed by the constant mass flow rate of the spray, $\dot{M}$ . Due to the movement of the spray operator, for example during wall spraying and/or the entrained airflow related to the spray process, the personal volume is exchanged by a constant airflow rate, $Q_{p}=V_{p}/T_{p}$ . Thereby $T_{p}$ represents the residence time of the spray mass in the personal volume. Subsequently the mass will pass into the far-field room volume, V_R_, where it is assumed to be instantaneously homogenized inside the entire volume. This causes a constant (near field) concentration of the sprayed substances (and solvents) inside the personal volume during the spraying time, $T_{s}$ .

Accordingly, for the (non-volatile) substance the concentration in the near field (Eq. 1) can be expressed as


(1) $$C_{\phi_{i},nf}=\frac{\dot{M}\phi_{i}}{Q_{p}}$$

and the concentration pattern in the far field (Eq. 2) taking into account the air exchange rate, Γ , is


(2) $$C_{\phi_{i},\mathrm{ff}}\left(t\right)=\frac{\dot{M}\phi_{i}}{V_{r}\Gamma }\{\begin{array}{cc} \left(1-e^{-\Gamma t}\right) & for\;t<T_{s}\\ \left(1-e^{-\Gamma T_{s}}\right)e^{-\Gamma \left(t-T_{s}\right)} & \;for\;T_{s}\leq t<T \end{array}$$

inside the entire room volume, $V_{R}$ , during the spraying time and post spraying time if the material is instantaneously mixed. Time integration yields the contributions to the time weighted average (TWA) mass concentration of the sprayed formulation in the personal and the room volume:


(3) $${\bar{C}}_{\phi_{i},nf}=\frac{\dot{M}\phi_{i}}{Q_{p}}.\frac{T_{s}}{T}$$

(4) $${\bar{C}}_{\phi_{i},\mathrm{ff}}=\dot{M}\phi_{i}.\frac{T_{s}\;}{V_{R}}.\frac{1}{\Gamma T}\left[\begin{array}{l} 1-\frac{1}{\Gamma T_{s}}\left(1-e^{-\Gamma T_{s}}\right)+\frac{1}{\Gamma T_{s}}\\ \left\{1-e^{-\Gamma \left(T-T_{s}\right)}\right\}\left\{1-e^{-\Gamma T_{s}}\right\}\; \end{array}\right]$$

The TWA of the exposure concentration is approximated by the sum of the two terms which is a good approximation for $T_{p}\ll T$ :


(5) $$\bar{C}\phi_{i}={\bar{C}}_{\phi_{i},nf}+{\bar{C}}_{\phi_{i},\mathrm{ff}}$$

In summary, the mandatory input parameters for the simple generic 2-box model used here are: the room volume, the air exchange rate, the spraying and exposure time, the mass flow rate of the sprayed liquid, and the mass fraction of the substance under consideration in the sprayed liquid. For the volumetric exchange flow rate, $Q_{p}$ , of the personal volume, a value of 100 m^3^/min is suggested as a fixed value. This value is larger than values found in the literature for stationary sources ranging up to 30 m^3^/min (8). The higher value has been chosen due to the movement of the sprayer and the forced airflow by air entrainment into the spray. For a value of 10 m^3^ for the personal volume $V_{p}$ , the volume flow rate of 100 m^3^/min corresponds to a residence time, $T_{p}$ , of the spray in the personal volume of 0.1 min.

#### 2.1.2 Refined generic 2-box spray model

The generic 2-box model (Eqs. 3–5) assumes that all sprayed amounts end up in the air with the source term quantified by the release rate of the substance. For volatile substances, this approach assuming (instantaneous) complete evaporation is sufficient taking into account air exchange. For a non-volatile substance, the approach is expected to be over-conservative since droplet evaporation and settling is not taken into account as well as the reduced airborne fraction F_A_ for surface spraying which is due to wall deposition.

For room spraying, a value of 1 is suggested for F_A_ considering that all sprayed mass is released to air. For surface spraying, the value is usually <1 and strongly depends on the details of the spray nozzle such as cross-sectional surface area, spray angle, and exit velocity on the spray as well as on the droplet spectrum generated in the spray process. These parameters may vary significantly for different spraying systems, and usually the operational parameters of the spraying system are not known in detail. A default value of 30% for the airborne fraction seems to be a reasonable worst-case for surface spray applications since spraying systems leading to higher overspray formation are unlikely to be used for this type of application considering that the intention is that the substance is on the surface and not off the target. Measurements by Schwarz, Koch (9) and estimations derived from a detailed wall impaction model presented in Hahn, Schwarz (10) support this assumption.

Droplet evaporation is determined by the solvent vapor pressure and settling depends on the (resulting) droplet size distribution. For exposure situations for which at least some generic information or assumptions on solvent vapor pressure and droplet size distribution are available, a refined version of the generic 2-box model was developed. This refined 2-box model accounts for droplet maturation and settling by correction factors ξ and κ applied to the far field and near field contribution of the TWA concentration “Applying these correction factors to Eq. 5, results in the following Eq. 6.


(6) $${\bar{C}}_{\phi_{i},\mathrm{corr}}=\left({\bar{C}}_{\phi_{i},nf}.\xi +{\bar{C}}_{\phi_{i},\mathrm{ff}}.\kappa \right)\;.F_{A}$$

The correction factors were calculated using a more detailed analytical well stirred one compartment model applied to the personal volume and the room volume that takes into account the aerosol dynamics of droplet evaporation and droplet settling. The details of this analytical spray model are presented in the Supplementary material.

The correction factors to the simple generic 2-box model related to droplet evaporation and settling are given by


(7) $$\xi ={\bar{\tilde{C}}}_{\phi_{i},nf}/{\bar{C}}_{\phi_{i},nf}\text{ and }\kappa ={\bar{\tilde{C}}}_{\phi_{i},\mathrm{ff}}/{\bar{C}}_{\phi_{i},\mathrm{ff}}$$

where ${\bar{\tilde{C}}}_{\phi_{i},nf}$ and ${\bar{\tilde{C}}}_{\phi_{i},\mathrm{ff}}$ are the TWA concentrations calculated by the extended analytical model. In a comparison of Eq. 7 with Eqs 3, 4, it is obvious that the correction factors (ratios of two concentration values) are independent of the room volume and the mass flow rate of the spray liquid because both calculated concentrations depend in the same way on these parameters. Parameters primarily influencing the values of the correction factors are the droplet distribution, which can be described by a lognormal distribution with variable mass median droplet diameter $\left(MMD\right)$ and constant geometric standard deviation, $\sigma_{g}=1.8$ (11), and the vapor pressure of the solvent as well as the spraying and exposure times, $T_{s}$ and T . Further parameters are the air exchange rate, Γ , the settling height, $H_{s}$ , and the volume fraction of the non-volatile substances, $\hat{\phi }$ .

For the model runs to derive the correction factors, the following model input data were used: The solvent vapor pressures values were chosen in steps of 0.01, 0.1, 1, 10, 100, 10,000, and 10,000 Pa. The mass median droplet diameters of the spray droplet spectrum were chosen in steps of 10, 20, 40, 80, 160, 320, and 640 μm. The geometric standard deviation was set to a constant value of 1.8. The overall effect of settling losses that determine the correction factors for the far-field contribution depends on the time scales and on the air exchange rate. With large values for the air exchange rate, for example, mass losses by settling become less important compared to mass losses by ventilation. Therefore, the spraying time, $T_{s}$ , the exposure time, T and the air exchange rate, Γ , were chosen according to Table 1. Table 1 represents typical values for real life application of spray: short, medium and long spraying times as well as short and long post exposure times (per treated location). Far-field correction factors were calculated for 0, 10, and 20 h^−1^ for the exposure durations of 6 and 15 min.

**Table 1:** Values of spraying and exposure time used for calculation of the correction factors.

$T_{s}$ [min]	T [min]	$T/T_{s}$	Γ [1/h]
1	6	6	0, 10, 20
10	15	1.5	0, 10, 20
10	60	6	0, 5, 10, 20
60	65	1.08	0, 5, 10, 20

The value of the settling height was set to 3 m as a typical room height. The effect of settling on the TWA after evaporation of the solvent is influenced by the volume fraction of all non-volatile compounds in the application solution. Here a value of 0.01 was chosen to be conservative. Larger values of the volume fraction would lead to smaller values of the correction factor, i.e., lower concentration because the size (MMD) of the matured aerosol (after solvent evaporation) is larger resulting in higher settling losses. Values of the volume fraction of non-volatiles smaller than 0.01 seemed unlikely in practice, as other impurities add to the background concentration of non-volatiles in the final spray solution. For example, the formulation does not often contain only one non-volatile substance. In addition, concentrated solutions of the formulation containing the non-volatile compound [for example quaternary ammonium compound (QACs)] are typically diluted with tab water, and tab water usually contains other non-volatile compounds such as salts, e.g., a medium water hardness of 14° is equivalent to 0.25 g/L calcium carbonate. A value of 1,000 kg/m^3^ was assumed as default for the solvent and substance densities.

The vapor pressure of the solvent (which can be a mixture of different compounds) and the droplet spectrum may cover a range of values and are not always readily available. Therefore, a categorization of the parameters was carried out. Three vapor pressure classes, 1–3 (class 1: ≤ 10 Pa, class 2: 10–1,000 Pa, class 3: ≥ 1,000 Pa), and two size classes (fine spray: MMD 10, 20, 40, 79 μm and coarse spray: MMD 80, 160, 320, 640 μm) were chosen. The fine spray is representative for room spraying using propellant aerosol cans or fogging systems as particles are intended to have a long residence time in the air. High pressure spraying typically generates fine droplets whereas coarse sprays result from spray nozzles operated at low pressure (< 6 bar). In order to adjust the generic 2-box model using the restricted information on vapor pressure and droplet size, the mean value, $\bar{\kappa }$ , of the κ -values belonging to the parameters that determine the vapor pressure and droplet size class was calculated. This was done for all scenario parameters listed in Table 1, resulting in 3 × 2 × 14 = 84 values for $\bar{\kappa }$ for the far-field correction. The calculated values are listed in Table 2.

**Table 2:** Mean values of the near- and far-field correction factors $\bar{\xi }$ and $\bar{\kappa }$ .

$T_{s}$ [min]	T [min]	Γ [1/h]	1 fine	1 coarse	2 fine	2 coarse	3 fine	3 coarse
			Mean far-field correction factor, $\bar{\kappa }$					
1	6	0	0.38	0.03	0.61	0.09	0.69	0.16
		10	0.40	0.03	0.61	0.10	0.70	0.17
		20	0.41	0.04	0.62	0.10	0.71	0.18
10	15	0	0.35	0.02	0.59	0.08	0.66	0.13
		10	0.38	0.03	0.60	0.09	0.68	0.15
		20	0.40	0.04	0.62	0.10	0.70	0.17
10	60	0	0.27	0.01	0.45	0.04	0.48	0.05
		5	0.32	0.02	0.54	0.06	0.59	0.10
		10	0.36	0.03	0.58	0,08	0.65	0.13
		20	0.40	0.04	0.61	0.10	0.70	0.17
60	65	0	0.29	0.01	0.49	0.04	0.52	0.06
		5	0.33	0.02	0.55	0.07	0.61	0.10
		10	0.36	0.03	0.59	0.08	0.66	0.13
		20	0.40	0.04	0.62	0.10	0.70	0.17
			Mean near-field correction factor, $\bar{\xi }$					
0.1	0.1	0	0.65	0.34	0.73	0.36	0.84	0.43

The numbers 1, 2, and 3 denote the vapor pressure classes; the expressions “fine” and “coarse” characterize the droplet size distribution.

The near field correction factors, ξ , were calculated for one scenario only. It was characterized by a droplet residence time inside the personal volume of 0.1 min. The residence time was obtained from the air exchange flow rate of 100 m^3^/min and an assumed personal volume of 10 m^3^. A total of 3 × 2 × 1 = 6 mean values, $\bar{\xi }$ , of the mean near field correction factor was calculated (Table 2).

#### 2.1.3 Generic 2-box spray model using release fraction

The default value for the airborne release fraction during surface spraying of 30% is probably too conservative when treating flat surfaces such as the walls and the floor of the room. It may be justified that the spray partly passes the surfaces to be treated such as spraying on industrial appliances with structured surfaces (tubes, grids) or carrying out disinfection tasks in stables.

An alternative to select this value is to use measured values of the airborne release fraction of (non-volatile) compounds obtained in control chamber experiments (9, 12–14). The airborne release fraction takes into account overspray formation and settling losses in the immediate vicinity of the spray nozzle. In the study by Schwarz, Koch (9), the airborne release fraction of non-volatile substances in the inhalable aerosol was roughly classified into $F_{A}=0.01$ for flat fan and hollow cone spray nozzles operated at low (1–3 bar) and high (<10 bar) pressures and $F_{A}=0.1$ for handheld pump sprays. It was measured under conditions of realistic application for the spray technology.

### 2.2 Workplace measurement data

The model and its two refinement options were evaluated by comparing model calculations with available experimental measurement data for typical workplaces. Different sources of suitable workplace measurements data have been identified. Measurement data are available from BAuA reports [F1702 (15); F2137 (16), F2366 (9)]. Data from F2137 (16) were already used for evaluation of the SprayExpo model. In addition, suitable data were available from the Biocides Human Health Exposure Methodology (17): Spraying Model 2 contains data from HSE (18) and Spraying Model 10 contains data from TNO report V3806 (19). Further data were published for insect sprays by Berger-Preiss, Koch (20), which were also used for evaluation of the performance of ConsExpoWeb on modeling consumer exposure to spray products (21). For the modeling exercise, the experimental conditions described in the identified studies were coded for the input parameters required and simulated using the described models (see Supplementary material).

In total for evaluation of the screening models, 34 scenarios were used from the BAuA reports [F1702 (15), F2137 (16), F2366 (9)] representing different sprayed masses, room volumes, and spraying times as well as different application types (surface or room spraying) and mass median diameters (MMD) of the sprayed aerosols. The use areas covered antifouling, pest control, wood protection, stored product protection as well as disinfection of tables, walls, or pool sides or treatment of animal housings. Room volumes ranged from relatively small rooms with 13.3 m^3^ to very large with 10,700 m^3^. For the ventilation rates, specific values were available for three scenarios. In the cases where information was missing, a rough estimation was made: for antifouling scenarios, an air exchange rate of 10 h^−1^ has been assumed, for animal housings a rate of 1 h^−1^ due to open doors, and for all other scenarios a rate of 0.6 h^−1^. Spraying time was usually equal to exposure time (sampling time) and ranged from 4 to 103 min. Exceptional cases are the table disinfection at which spraying time of 1 and 1.28 min were shorter than the exposure time of 4.5 and 4.45 min, respectively.

Data from HSE (18) are available including 13 scenarios with measured data for inhaled exposure during spray application (in total 20 scenarios considering dermal exposure data). Spraying indoors ranged from small-scale domestic to large-scale applications. The room volume spanned a high variability from living rooms to church halls, but specific values on room volumes and ventilation rates were not given. Spraying was done onto hard surfaces, and the direction was usually “around,” partly overhead and for some scenarios downwards. Besides spraying, half of the scenarios also included irrigation (injection into holes), but information on the fraction of irrigation on the whole process was not given. Spray pressures ranged from 320 to 1,050 kPa and spraying activity from 6 to 95 min. For the simulation, a worst-case assumption was used to cover uncertainties in the boundary conditions: all mass is sprayed not irrigated, particle size class is fine, and ventilation is low (0.6 h^−1^).

In the TNO report V3806 (19), in total 16 scenarios were given on surface spraying for pest control in different areas such as private home, chicken stable, transit store, and restaurant. The room volumes varied from 192 to 40,728 m^3^ and the treated surface (no input parameter) from 64 to 5,556 m^2^. Ventilation was given qualitatively with no ventilation, natural ventilation, or mechanical ventilation, so that for modeling 0, 0.6, and 10 h^−1^ were selected, respectively. Generally, equipment with a shoulder strap was used with <3 bar. However, one scenario comprised equipment with >3 bar and another scenario with equipment for fogging. Different temperatures were given for each scenario which had no impact on the modeled results using the described models, as the temperature range of 7.5 to 20.1°C had no influence on the assignments on vapor pressure classes of the solvent or non-volatile substance. The amount sprayed and the measured concentration of the substance of interest in the spray tank were specified for each scenario. Sampling time was used for spraying time and exposure time, as directly linked to the measured TWA given. Overall, although some minor uncertainties regarding ventilation or particle sizes existed, sufficient information on the boundary conditions were available for these measurements.

Measured data for application of 5 different insect sprays were presented in Berger-Preiss, Koch (20), and each product consisted of 2–3 non-volatile substances. The insect sprays were applied all for room spraying with a fine aerosol (MMD < 40 μm), and each in three different time scales [variation in spraying (10 s to 2 min) and exposure time (2.2 –to 60 min)] as well as in sprayed amount (9.5 to 189.2 g) resulted in 15 different scenarios. Only the substance with the highest content was selected for the modeling exercise for each scenario and was compared to the measured value of this compound. Room volume was relatively small (about 40 m^3^), and a low ventilation rate of 0.6 h^−1^ was presumed.

Finally, the modeled time-weighted average (TWA) air concentrations using the described models were compared with the measured air concentrations to evaluate the performance of the described models.

### 2.3 Statistical methodology

In recent publications, statistical parameters have been proposed and discussed to evaluate performance and accuracy of models (8, 22–24). However, no agreed standards exist (25). In the following, the ratio modeled/measured concentrations have been calculated for each workplace scenario. Based on these ratios, the percentage of the number of scenarios with ratio < 0.5, 0.5–10, 10–100, and > 100 has been derived.

## 3 Results

### 3.1 Correction factors

For practical application of the generic 2-box model and the refinement using correction factors, the latter should be calculated in advance. For this purpose, a series of model runs was performed with the analytical model covering the range of expected exposure scenarios (see section 2.1.2).

**Figure 2:**
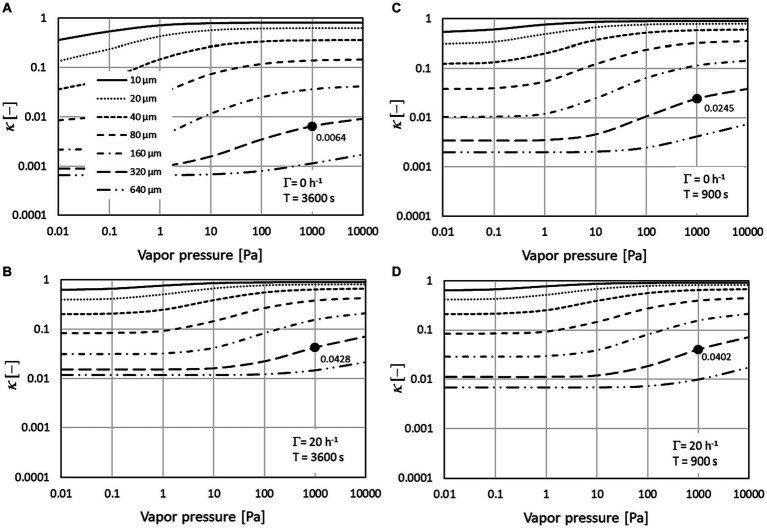
Correction factors for 10 min spraying duration (Ts) and overall exposure time (T) of 60 min **(A,B)** or 15 min **(C,D)**. Comparison between scenarios without air exchange **(A,C)** and an exchange rate of 20 h^−1^ **(B,D)**. The dots are the exemplary values for a solvent vapor pressure of 1,000 Pa and a droplet spectrum with MMD of 320 μm.

Figure 2 shows results calculated for the parameters of the second and third row of Table 1 representing spraying times of 10 min, which are typical for disinfection of surfaces inside a room. Parameters of the calculations varied with respect to the post exposure time (15 versus 60 min and the air exchange rate (0 versus 20 h^−1^). No correction to the far-field contribution of the generic 2-box model (Eq. 4) results in κ=1 . The smaller the κ -value, the larger the deviation of the concentration calculated with the analytical model related to mass losses from droplet settling compared to the generic 2-box model without refinements. The main parameter of influence on the κ -value is the MMD of the droplet spectrum. The droplet size dependence is reduced to high values of the air exchange rate. This is because the residence time of the substance is smaller and, therefore, also the time that the settling mechanism is effective. Please note, the main influence of the air exchange rate on the TWA concentration is already accounted for in the generic 2-box model.

The dots show exemplary results for a MMD of 320 μm and a solvent vapor pressure of 1,000 Pa. For the 60 min exposure time (a, b) a reduction of the correction factor can be observed from κ = 0.0428 for 20 h^−1^ to κ = 0.0064 for zero air exchange. Obviously this difference is reduced for the shorter exposure time of 15 min (c, d): κ = 0.0402 for 20 h^−1^ and κ = 0.0245 for 0 h^−1^. For the large Γ -value of 20 h^−1^ the influence of exposure time on κ is small ( κ = 0.0402 for T = 15 min and κ = 0.0428 for T = 60 min) since the mean residence time where settling is effective is 3 min for both scenarios.

Figure 2 also shows that, compared to the droplet spectrum, the dependence of κ on vapor pressure is smaller. This is because for values above 1,000 Pa, the regime for most of the solvents, κ is nearly independent of vapor pressure because solvent evaporation from the spray droplets is fast and the mass losses are determined by the residual dry aerosol. At the low end of the vapor pressure scale (<1 Pa), there is virtually no droplet evaporation, and mass losses are determined by the size of the spray droplets.

Generally the far-field correction factors are smaller for coarse sprays than for fine sprays. For water which is classified as vapor pressure class 3, the correction factors for the fine droplet spectrum vary between 0.48 and 0.70 and for the coarse droplet spectrum between 0.05 and 0.17. For short exposure times, the influence of the air exchange rate on the mean far-field correction factors is small. For 20-fold air exchange rate per hour, for example, the correction factors are independent of the spraying and exposure times since settling is active only during the residence time 1/Γ=3 min which is smaller than all the exposure time scale considered here. For the near field, the fine mode correction factor is close to 1 for the fine spectrum and about 0.4 for the coarse mode spectrum.

### 3.2 Comparison with workplace measurements

The model and its refinements were compared with monitoring results obtained at workplaces. In total, 78 measurements from different sources were used for comparison. The measured substances in the spray formulations were all non-volatile. Most of the solvents (mainly water) belonged to vapor pressure class 3, which was important for the maturation of the droplets and thus the correction factor used for the refined generic 2-box model. However, measurement data for the solvents were usually not available, so only the concentrations of the non-volatile substances were available for comparison.

#### 3.2.1 Data from BAuA reports

**Figure 3:**
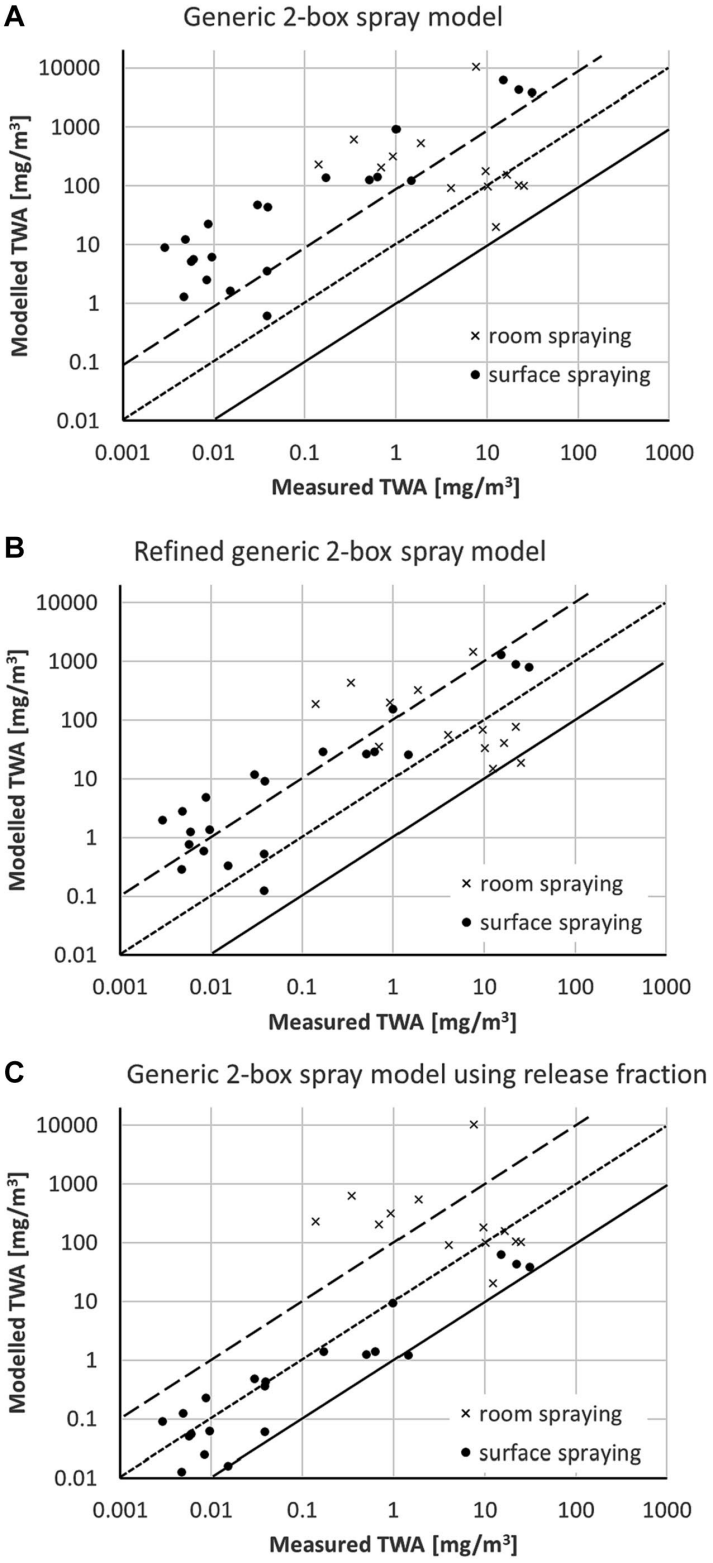
Comparison of modeled TWA values with measured data from the BAuA reports (15, 16, 9) using **(A)** the generic 2-box spray model, **(B)** the refined generic 2-box spray model, and **(C)** the generic 2-box spray model using release fraction; solid line represents 1:1-line, the dotted line a 10-fold, and the dashed line a 100-fold overestimation of the model.

The comparison of the modeled TWA with workplace data from the BAuA reports is shown in Figure 3 (15, 16, 9). For surface spraying, the generic 2-box spray model (Figure 3A) usually overestimates the measured TWA values by a factor of 100 and larger. Using the refined generic 2-box spray model (Figure 3B) reduces the conservatism of the model due to applying the correction factors $\bar{\kappa }$ and the default airborne fraction of 30%. However, the modeled TWA values for the surface spraying scenarios are mostly still at least a factor of 10 above the measured TWA values. This changes when the data-based classification of the airborne release fractions associated with the spray technology is used for $F_{A}$ in the generic 2-box spray model. The predictions for surface spraying become significantly less conservative with many of the scenarios falling within the range between the measured TWA and 10-fold above the measured TWA (Figure 3C).

For room spraying the situation is quite different. The modeled TWA values are above the measured TWA using the generic 2-box spray model (Figure 3A), but only a few scenarios are highly overestimated (> factor 100). Considering the settling of particles using the correction factors $\bar{\kappa }$ significantly reduces the conservatism of the model if coarse particles are present (refined generic 2-box spray model, Figure 3B). Using the airborne release fraction approach (Figure 3C) will not change the estimate in comparison to the generic 2-box spray model (Figure 3A), as for room spraying all sprayed liquid becomes airborne (airborne release fraction $F_{A}$ = 1).

**Figure 4:**
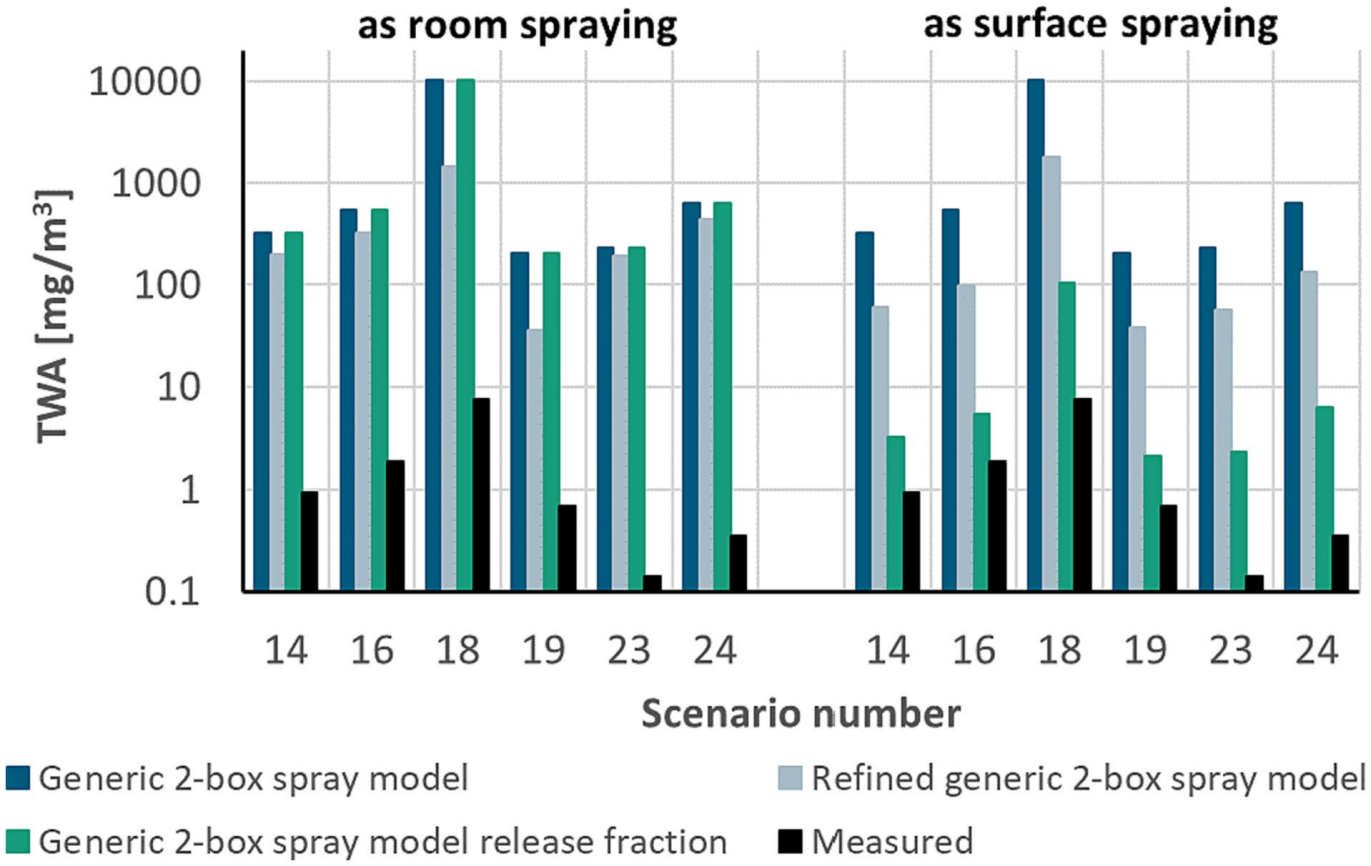
Comparison of modeled and measured TWA values for scenarios calculated for both room and surface spraying, as application type is unclear. For scenarios numbers and specific information, see Supplementary material.

The data points above the 1:100 line in Figures 3A,C represent stable disinfection and wood protection scenarios. In these scenarios not only flat surfaces are treated but also beams and grids, and thus parts of the spray pass the surfaces to be treated. The actual application type is consequently a mixture of room and surface spraying. Room disinfection with $F_{A}$ = 1 has been selected for these scenarios as a worst-case assumption. However, a significant part of the spray is expected to be on the surface, so that $F_{A}$ is actually <1. Using for these scenarios surface spraying (Figure 4) would shift the model closer to the measured data if the airborne release fraction approach is used with an $F_{A}$ = 0.01. The TWA values calculated by the refined generic 2-box spray model are less conservative than the generic 2-box spray model but are still apparently higher than the release fraction approach. For these specific scenarios and measurements, the selection of surface spraying instead of room spraying is a refinement option, is still conservative, and seems to be more appropriate than room spraying.

#### 3.2.2 Data from model 2 of biocides human health exposure methodology

**Figure 5:**
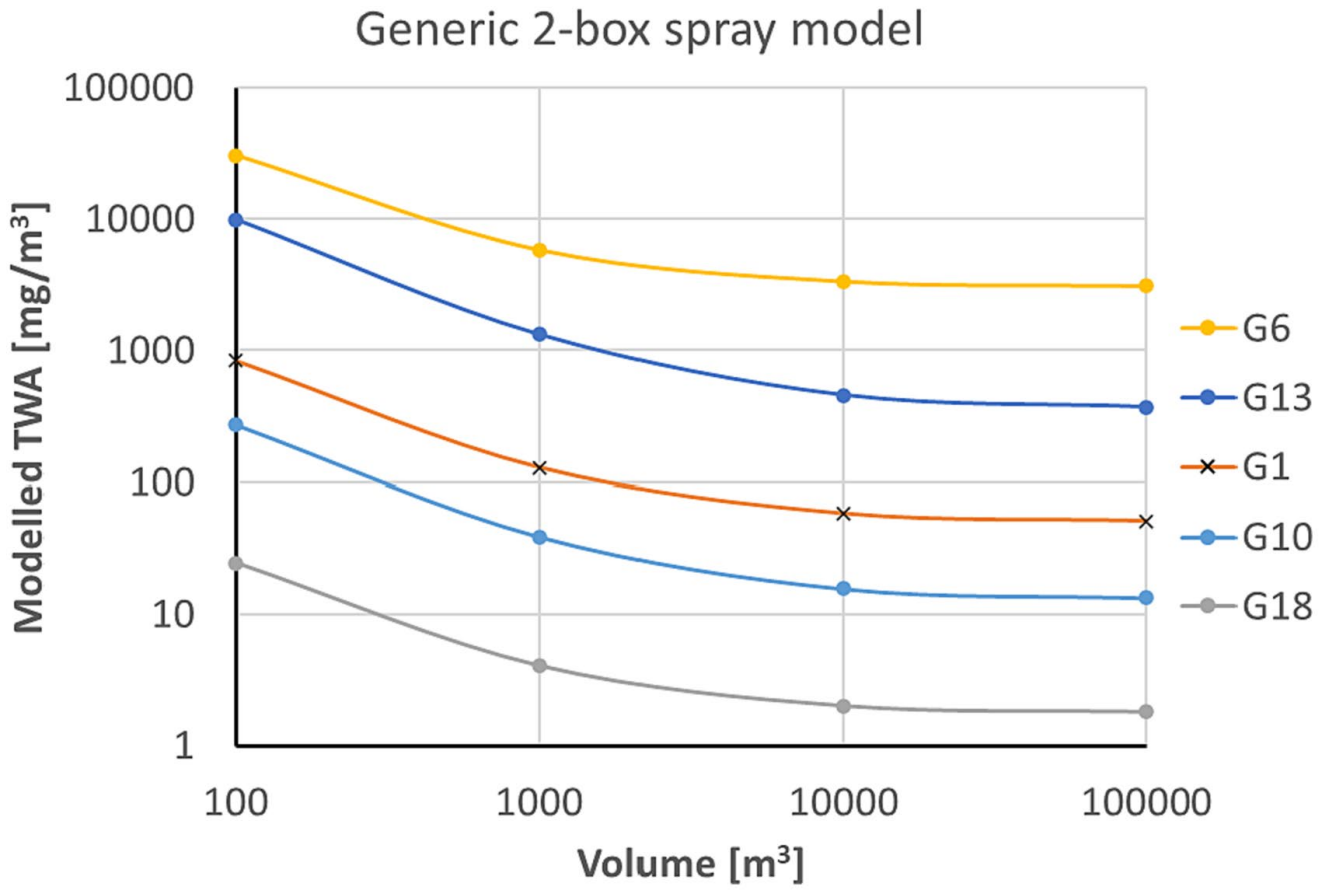
Dependence of modeled TWA values from room volume used in the generic 2-box spray model for exemplary scenarios from HSE (18).

**Figure 6:**
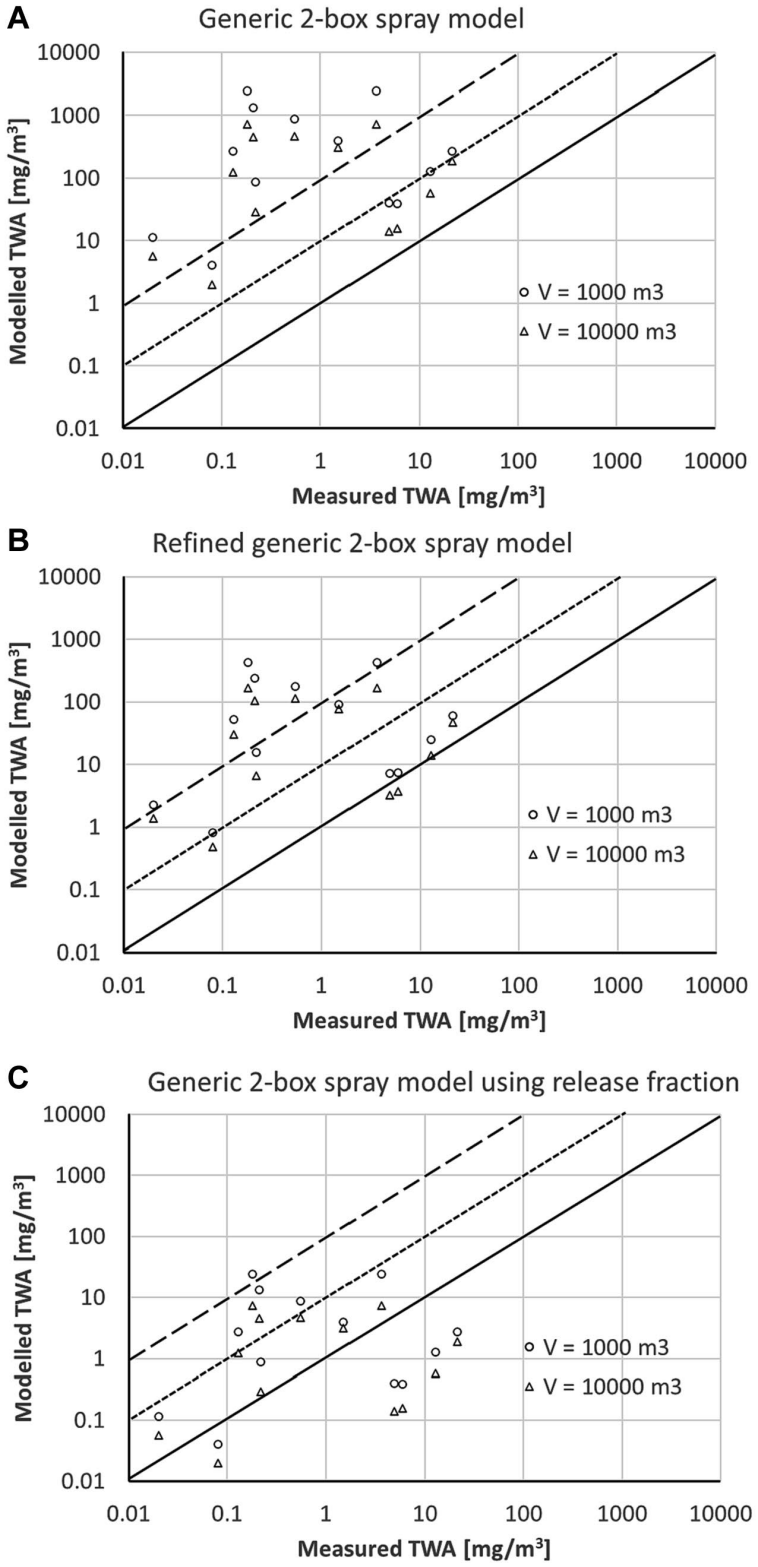
Comparison of modeled TWA values with measured data from HSE (18) using **(A)** the generic 2-box spray model, **(B)** the refined generic 2 box spray model, and **(C)** the generic 2-box spray model using release fraction; solid line represents 1:1-line, the dotted line a 10-fold, and the dashed line a 100-fold overestimation of the model.

For the workplace data from Garrod, Rimmer (18), all scenarios were coded as surface spraying with a particle size “fine.” In the publication of Garrod, the room volume of each workplace was not specified. However, the uncertainty of the room volume was not expected to have much impact. Figure 5 shows the dependency of the modeled TWA from the input parameter room volume. With increasing room volume, the TWA converges to a value which is determined by the concentration in the near field (personal volume). This clearly shows the strength of the 2-box model. Subsequently, simulations were performed for all scenarios using two different room volumes (1,000 m^3^ and 10,000 m^3^), which are probably too high, for example, for the sampled scenarios in living rooms but too small for example for the sampled scenarios in a chapel. The comparison of modeled TWA with the measured workplace data is shown in Figure 6. For both assumed room volumes, the modeled TWA are often at least by a factor of 100 higher than the measured TWA values using the generic 2-box spray model (Figure 6A). The TWA is more realistic but in most cases still higher than the measured value using the refined generic 2-box spray model (Figure 6B). The third model using the release fraction of 0.01 with the generic 2-box spray model results in TWA values significantly below the measured values for some scenarios (Figure 6C). This indicates that the airborne release fraction of 0.01 may not be appropriate for these situations. However, information on some relevant boundary conditions are not available, resulting in a high uncertainty of the input parameters and thus also in an uncertainty of the modeled TWA.

#### 3.2.3 Data from model 10 of biocides human health exposure methodology

**Figure 7:**
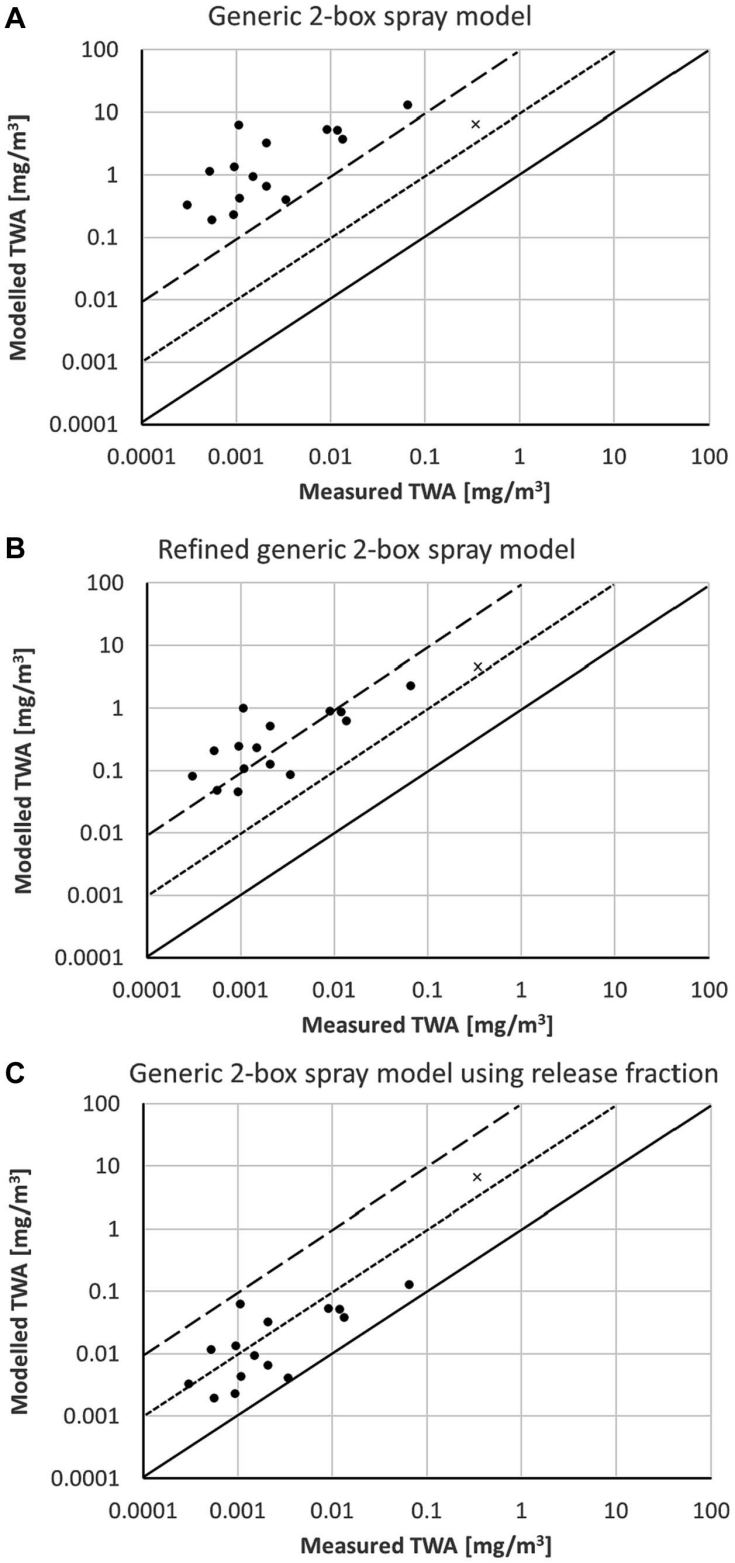
Comparison of modeled TWA values with measured data from TNO (19) using **(A)** the generic 2-box spray model, **(B)** the refined generic 2 box spray model, and **(C)** the generic 2-box spray model using release fraction; solid line represents 1:1-line, the dotted line a 10-fold, and the dashed line a 100-fold overestimation of the model; all scenarios surface spraying with one exemption for fogging.

The TNO data were mostly coded as surface spraying as well (19). One scenario is for fogging application, and thus it cannot be assumed that deposition on surface during application is relevant. This scenario was assigned as a worst-case approach to application type room spraying. The comparison of modeled TWA with the workplace data is shown in Figure 7. Again the generic 2-box spray model (Figure 7A) usually highly overestimates the measured TWA by a factor of >100 due to neglecting deposition on the surfaces. Applying the correction factor (Figure 7B) will reduce the modeled TWA but is still conservative. Using the airborne release fraction approach in the generic 2-box spray model results in an estimation nearest to the 1:1 line.

#### 3.2.4 Data for insect sprays

**Figure 8:**
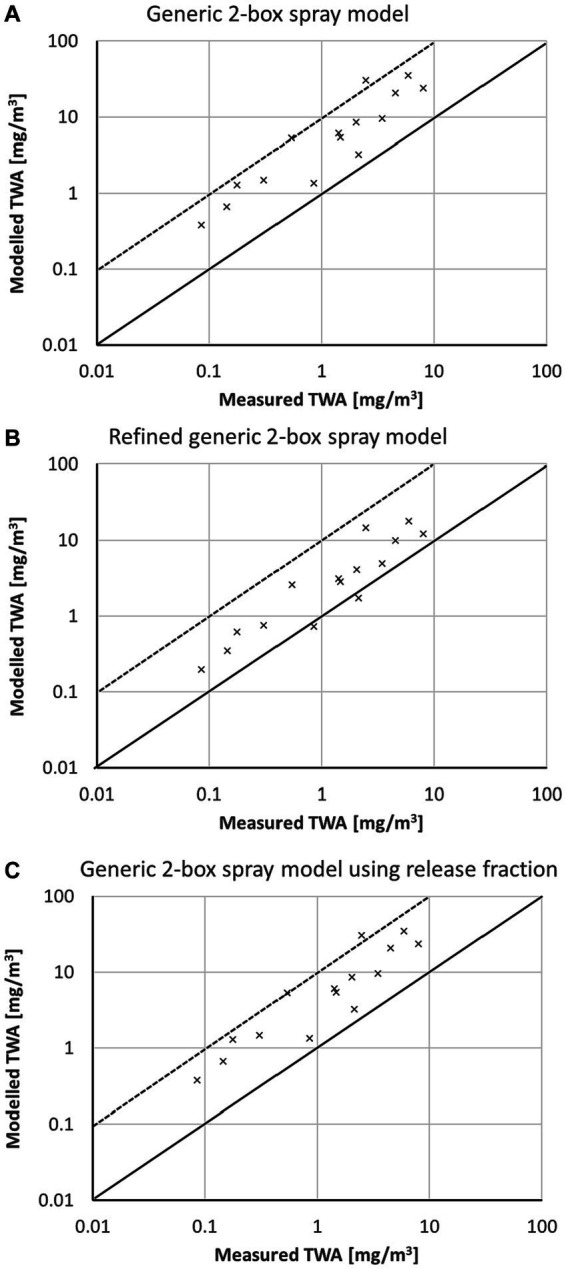
Comparison of modeled TWA values with measured data for insect sprays (20) using **(A)** the generic 2-box spray model, **(B)** the refined generic 2 box spray model, and **(C)** the generic 2-box spray model using release fraction; solid line represents 1:1-line, the dotted line a 10-fold overestimation of the model; all scenarios room spraying.

The measurements of Berger-Preiss, Koch (20) were solely simulated as room spraying with a particle size class of “fine.” The comparison of modeled TWA with measured workplace data is shown in Figure 8. For room spraying all sprayed liquid becomes airborne ( $F_{A}$ = 1), and thus there is no difference between Figures 8A,C. The modeled data using the generic 2-box spray model are usually up to a factor of 10 above the 1:1 line. The refined generic 2-box spray model (Figure 8B) results in slightly reduced modeled TWA values, which is due to the consideration of the settling and which is only marginal for fine particles.

## 4 Discussion

In this article, a generic 2-box spray model is presented for screening purposes in order to estimate the exposure during spraying activities. In addition, approaches are suggested to refine the model outcome without using higher tier tools. In recent publications, criteria has been proposed and discussed to evaluate performance and accuracy of models which are based on the ratio modeled/measured concentrations (8, 22–24). However, these criteria seem to be too ambitious for screening models, as such screening models (tier 1 models) should represent the best possible compromise between accuracy and simplicity, and, therefore, often the modeled estimates are significantly higher than the actual exposure. For this reason an underestimation was assigned to a ratio < 0.5, an accurate estimation for ratio 0.5–10, an overestimation to ratio 10–100, and a high overestimation to ratio > 100. An overview of the statistical data evaluation is given in Table 3.

**Table 3:** Statistical data using the 2-box spray models.

		Generic	Refined generic	Generic release fraction
BAuA data (15, 16, 9); number of entities 34; different application types (surface and room spraying; different particle size classes)	Underestimation (T/M < 0.5)	0.0%	0.0%	0.0%
	Accurate (0.5 < T/M < 10)	14.7%	20.6%	61.8%
	Overestimation (10 < T/M < 100)	14.7%	35.3%	20.6%
	High overestimation (T/M > 100)	70.6%	44.1% ^a^	17.6% ^a^
HSE data (18); number of entities 13; surface spraying, uncertainty regarding room volume (*V* = 1,000 m^3^), and particle size class (fine)	Underestimation (T/M < 0.5)	0.0%	0.0%	30.8%
	Accurate (0.5 < T/M < 10)	15.4%	30.8%	38.5%
	Overestimation (10 < T/M < 100)	23.1%	23.1%	23.1%
	High overestimation (T/M > 100)	61.5%	46.2%	7.7%
TNO data (19); number of entities 16; surface spraying (and one room spraying/fogging); uncertainty regarding particle size classes (mostly coarse used)	Underestimation (T/M < 0.5)	0.0%	0.0%	0.0%
	Accurate (0.5 < T/M < 10)	0.0%	0.0%	62.5%
	Overestimation (10 < T/M < 100)	6.3%	62.5%	37.5%
	High overestimation (T/M > 100)	93.8%	37.5%	0.0%
Insect sprays (20); number of entities 15; room spraying, particle size class fine	Underestimation (T/M < 0.5)	0.0%	0.0%	0.0%
	Accurate (0.5 < T/M < 10)	93.3%	100%	93.3%
	Overestimation (10 < T/M < 100)	6.7%	0.0%	6.7%
	High overestimation (T/M > 100)	0.0%	0.0%	0.0%

T / M = ratio tool estimate to measured TWA; a = using surface spraying will reduce high overestimation.

All three models give conservative results, as almost all modeled values are higher than the measured values. The prediction of the generic 2-box spray model highly overestimates the measurements for the considered scenarios in approximately 60% of the cases by more than a factor of 100. However, performance of the generic 2-box spray model is excellent for room spraying, and the modeled value is usually maximum a factor 10 higher than the measured value. Exemptions are the scenarios for which application type is not an unambiguous assignment, and thus room spraying is used as a worst-case assumption. If detailed information on the application type and equipment are available, the selection of surface spraying and measurement or defining the airborne release fraction may be an appropriate refinement option. However, if only limited information on the scenario and equipment is available, the default of 30% airborne fraction or even room spraying should be selected as a worst-case approach to avoid underestimation.

Underestimation is generally not observed for the generic 2-box spray model which is not surprising as the model assumes that all sprayed amount is airborne regardless of surface or room spraying. In addition, TWA values during spraying are determined by the concentration in the near field (personal volume) so that by using the 2-box model approach the influence of the dilution within the room volume has minor impact and an underestimation is not expected.

For surface spraying the overestimation in the generic 2-box spray model is primarily based on neglecting intended deposition of spray on the treated surface. Taking settling into account, which is implemented in the refined generic 2-box spray model, reduces the conservatism. Although settling is dependent on the particle size, it is not necessary to know the particle size distribution in detail but it is sufficient to rather have a rough classification into fine or coarse spray. Further refinement option addresses the airborne fraction which is important for surface spraying only. An airborne fraction of 30% is suggested as a default in the refined generic 2-box model and at the same time only fine particles as airborne are considered. Using the experimentally determined airborne release fractions, which indirectly reflect the complex characteristics of the spraying equipment and which are much smaller than the suggested default value, results in the least conservative and thus most accurate prediction of measured concentrations. In more than 60% of the cases, the modeled value for the spraying scenarios is then below a factor of 10 above the measured values. It is worth mentioning that for the generic 2-box model based on airborne release fractions no information on the droplet size distribution is required.

Underestimation has been observed for the HSE data (18) when using the airborne release fraction approach on the generic 2-box model. It seems that the used airborne release fraction is not appropriate to all these scenarios and some are rather similar to room spraying. However, in this case, the coding of the scenarios has a high uncertainty due to missing information on room volume, ventilation, application, and equipment. As a worst-case approach, room spraying should be selected if sufficient information is not available. However, for the BAuA data, it has been demonstrated that room spraying could also result in high overestimation and surface spraying considering the airborne release fraction is the better choice. In case of doubt, the airborne release fraction should be measured experimentally.

The evaluation of the performance of the presented screening models has only taken the measurement of non-volatile substances into consideration so far. However, the generic 2-box spray model approach, at which all sprayed liquid becomes airborne, is principally also applicable to volatile substances. Even if volatile substances deposit on the surface, they will become airborne by evaporation. For this reason, assuming that all substances are after spraying, airborne is a reasonable worst-case assumption. As only limited data are available for volatiles during spraying, this model domain can hardly be evaluated quantitatively. In Hahn, Schwarz (10) some information on volatiles are presented that supports the expectation that the presented screening approach may be conservative for volatile substances. A potential refinement for volatiles substances may only be possible using higher tier tools such as SprayEva (26). In addition, the presented model is evaluated so far mostly for indoor application only. As mentioned earlier, often spraying activities are applied outdoors (e.g., pesticides). For outdoor environments, the far field volume will be large, and additional distribution processes have to considered such as wind speed and direction. Wind will have an influence on the mass flow out of the personal volume but could also be directed into the personal volume which makes a prediction of the inhalable exposure more complex. Adaptation of the model and maybe different default input parameters may be required to be applicable to outdoor processes without limitations.

As mentioned above, a review of available models suitable to assess exposure during spraying activities is given in Hahn, Meyer (2). For example ECETOC TRA, ART or Stoffenmanager^®^ provide approaches to predict exposure during spraying activities. The majority of the available models are based on empirical data such as the TNsG spraying models [Biocides Human Health Exposure Methodology (17)]. Two datasets (HSE, TNO) provided by the TNsG spraying models have been used for evaluation of the presented screening models. The available mass-balance models (SprayExpo and ConsExpo) are regarded as higher tier models, as they need detailed information on the exposure situations such as information on particle size distribution. A comparison between the different models is beyond the scope of this publication, but there is a need for simple model approaches (6, 7).

ConsExpoWeb contains two model approaches: an instantaneous release model as screening and the more sophisticated spraying model mentioned above. The first one is similar to the presented generic 2-box spray model and considers the released mass, the weight fraction of the compound, the room volume, the exposure duration, and the ventilation. However, all the released amount is instantaneously released and homogenized in the air and does not consider the spraying time. In addition, it is based on a well-mixed room concept which is sufficient for small rooms usually presenting consumer exposure. However, for workplaces, often larger rooms are more typical, and thus a 2-box model concept seems to be more appropriate.

Several authors presented 2-box models. i.e. based on near field and far field (NF/FF) approaches (24, 27–34). Most of these approaches are applied to volatile substances which are evaporating from a source within the near field. The concept has been applied to spraying as well (29, 30), whereas Hofstetter, Spencer (29) concentrated on volatile compounds only. Critical parameters for the NF/FF model are the size of the near field (24) or the mass flow between near field and far field. Mass flow rates between near field and far field have been reported for several indoor environments in the range between 0.24 and 30 m^3^/min (8). The higher value of 100 m^3^/min has been proposed (see section 2.1.1) due to the movement of the sprayer and the forced airflow by air entrainment into the spray. Usually, near-field volumes of less than 1 to 25 m^3^ are suggested in literature (often 2x2x2 = 8 m^3^). A medium volume for the near field of 10 m^3^ corresponds for the proposed mass flow of 100 m^3^/min to a residence time of 0.1 min in the personal volume.

A 2-box model is also available in the IHMOD™ Tool published by AIHA. However, it is not developed specifically for spraying activities. For this reason, it considers the mass generation but does not consider the sink by settling or the deposition on the treated surface. This is also not considered in the presented generic 2-box spray model, but it is considered by the correction factors used for the refined generic 2-box spray model. In addition, the airborne release fraction approach considers the fraction which will adhere on the treated surface in the case of surface spraying as well as settling losses in the immediate vicinity of the treated area.

For the screening models (tier 1 approach) which are presented here, only easily obtainable input information is required. These are the room volume, the air exchange rate, the spraying and exposure time, the mass flow rate of the sprayed liquid, and the mass fraction of the substance under consideration in the sprayed liquid. For the refinements only information is required about the application type, i.e., surface or room spraying, and the vapor pressure class of the solvent. Additional refinement is possible if measured airborne release fraction is available or at least information about equipment which justifies the selection. If more information is available such as, for example, a detailed characterization of the spray droplet spectrum, higher tier models can be used as, for example, the analytical approach presented in the Supplementary material, SprayExpo (16, 35), and SprayEva (26).

The presented screening models can be regarded as a stage of extension for the ConsExpoWeb instantaneous release model considering the spraying time and the two-box model concept or for the AIHA model considering spraying activities and processes. Ultimately, the presented screening models expands the possibilities to use modeled data in regulatory authorization processes.

Although spraying has several advantages, the sprayed substances will become airborne and inhalable. As a result, several diseases are induced by these (workplace) activities which has been discussed, for example, by Clausen, Frederiksen (4). To prevent, control, and avoid these diseases, occupational health practitioners and exposure and risk assessors can make use of the presented generic 2-box model as a possible addition to workplace measurements. The model can be used for a first estimate to determine where at the workplaces concern about human health is expected, where more information is necessary (higher tier modeling and measurements), or where risk mitigation measures are needed. In comparison to the higher tier models, only easily obtainable input information is required. The generic 2-box model usually produces conservative exposure estimates. Thus, if the results of a risk analysis indicate that adverse health impacts are likely, the refinement options based on correction factors and measurement of release fractions provide an alternative to considering burdensome risk mitigation measures. The model can also be used to evaluate the impact of varying mass fraction, MMD, etc., in order to make recommendations for safe and sustainable by design (36, 37) products and systems, for example by altering the design of a spraying device and scenario. In consequence, the model will help to realize and control adverse human health effects during spraying of (corrosive) chemicals, which are often associated with a high inhalation burden.

## 5 Conclusion

The presented screening model is intended to be a simple introduction to exposure modeling of spraying activities, which also allows more refined estimates with slight adjustments. The model approaches using generic input parameters allow a conservative prediction of exposure concentrations for spray applications. However, the over-prediction of measured concentrations is quite large in particular for surface spraying due to significant overestimation of the airborne fraction. This can be reduced by using correction factors or the concept of airborne release fractions in which overspray formation and early spray aging is determined experimentally and categorized in view of the spray technology used. It would be worthwhile to enlarge the database of airborne release fractions and refine the categories in view of the specific scenarios and spray technologies. Combining this data set with the generic 2-box spray model could be a practical tool for conservative exposure prediction.

Overall, these screening models will complement the available models to assess spraying activities at workplaces. We have shown a way to replace necessary detailed technical information about the spray equipment (e.g., particle size distribution, nozzle information) with simple measurements or extraction of results from more complex modeling. Depending on the methodology used, different accuracies can be achieved.

## Data availability statement

All contributions presented in the study are included in the article/Supplementary material, further inquiries can be directed to the corresponding author.

## Author contributions

SH: Conceptualization, Formal analysis, Investigation, Project administration, Visualization, Writing – original draft. KS: Formal analysis, Methodology, Writing – review & editing. NN: Formal analysis, Software, Writing – review & editing. JS: Conceptualization, Project administration, Writing – review & editing. JM: Conceptualization, Supervision, Writing – review & editing. WK: Conceptualization, Formal analysis, Methodology, Visualization, Writing – original draft.

## References

[1] ISES Europe (2022). Europe exposure model inventory - worker, Version 1.0. Available at: https://ises-europe.org/exposure-platform/data-and-information-sharing

[2] Hahn S Meyer J Roitzsch M Delmaar C Koch W Schwarz J. Modeling exposure by spraying activities—status and future needs. Int J Environ Res Public Health. (2021) 18:7737. doi: 10.3390/ijerph18157737, PMID: 34360034 PMC8345348

[3] Marquart H Schneider T Goede H Tischer M Schinkel J Warren N. Classification of occupational activities for assessment of inhalation exposure. Ann Occup Hyg. (2011) 55:989–1005. doi: 10.1093/annhyg/mer072, PMID: 21926067

[4] Clausen PA Frederiksen M Sejbaek CS Sorli JB Hougaard KS Frydendall KB. Chemicals inhaled from spray cleaning and disinfection products and their respiratory effects. A comprehensive review. Int J Hyg Environ Health. (2020) 229:113592. doi: 10.1016/j.ijheh.2020.113592, PMID: 32810683

[5] GBD 2016 Occupational Risk Factors Collaborators. Global and regional burden of disease and injury in 2016 arising from occupational exposures: a systematic analysis for the global burden of disease study 2016. Occup Environ Med. (2020) 77:133–41. doi: 10.1136/oemed-2019-106008, PMID: 32054817 PMC7035694

[6] Schlüter U Arnold S Borghi F Cherrie J Fransman W Heussen H. Theoretical background of occupational-exposure models—report of an expert workshop of the ISES Europe working group “exposure models”. Int J Environ Res Public Health. (2022) 19:1234. doi: 10.3390/ijerph19031234, PMID: 35162257 PMC8834988

[7] Schlüter U Meyer J Ahrens A Borghi F Clerc F Delmaar C. Exposure modeling in Europe: how to pave the road for the future as part of the European exposure science strategy 2020–2030. J Expo Sci Environ Epidemiol. (2022) 32:499–512. doi: 10.1038/s41370-022-00455-4, PMID: 35918394 PMC9349043

[8] Ribalta C Lopez-Lilao A Fonseca AS Jensen ACO Jensen KA Monfort E. Evaluation of one- and two-box models as particle exposure prediction tools at industrial scale. Toxics. (2021) 9:201. doi: 10.3390/toxics9090201, PMID: 34564352 PMC8471509

[9] Schwarz K Koch W Günther F Schade C Göen T Schäferhenrich A. Human exposure to biocidal products: Measurement of inhalation and dermal exposure during the application of biocide foams. report BAuA, project F 2366. Dortmund: Bundesanstalt für Arbeitsschutz und Arbeitsmedizin (2022).

[10] Hahn S Schwarz K Nowak N Koch W Schwarz J Meyer J. Advancement and connection of modeling approaches for estimating inhalation exposure during spray applications. Report BAuA, project F 2492. Dortmund: Federal Institute for Occupational Safety and Health (BAuA) (2024).

[11] Kooij S Sijs R Denn MM Villermaux E Bonn D. What determines the drop size in sprays? Physical Review X. (2018) 8:031019. doi: 10.1103/PhysRevX.8.031019

[12] Delmaar JE Bremmer HJ. The Consexpo spray model - Modeling and experimental validation of the inhalation exposure of consumers to aerosols from spray cans and trigger sprays. Contract No.: Report 320104005/2009.

[13] Schwarz K Koch W. Thoracic and respirable aerosol fractions of spray products containing non-volatile compounds. J Occup Environ Hyg. (2017) 14:831–8. doi: 10.1080/15459624.2017.1335403, PMID: 28609222

[14] Loven K Isaxon C Wierzbicka A Gudmundsson A. Characterization of airborne particles from cleaning sprays and their corresponding respiratory deposition fractions. J Occup Environ Hyg. (2019) 16:656–67. doi: 10.1080/15459624.2019.1643466, PMID: 31361572

[15] Koch W Berger-Preiss E Boehncke A Könnecker G Mangelsdorf I (2004). Workplace exposure from the use of biocidal products - part 1. Inhalation and dermal exposure data for the spray application of liquid biocidal products. Report BAuA, project F 1702.

[16] Koch W Behnke W Berger-Preiss E Kock H Gerling S Hahn S. Validation of an Edp assisted model for assessing inhalation exposure and dermal exposure during spraying processes. Report BAuA, project F2137. Dresden: Fraunhofer Gesellschaft (2012).

[17] ECHA (2015). Biocides human health exposure methodology. Available at: https://echa.europa.eu/documents/10162/992289/bpr_exposuremethodbiochh_en.rtf/17e40d4c-5f48-4e12-952b-5372bfe2403c?t=1444729148304

[18] Garrod AN Rimmer DA Robertshaw L Jones T. Occupational exposure through spraying remedial pesticides. Ann Occup Hyg. (1998) 42:159–65. doi: 10.1016/S0003-4878(98)00006-4 9684556

[19] de Cock JS van Drooge H. Field study on occupational exposure during spraying of biocidal products by Pest control operators using deltamethrin and (ß)-cyfluthrin. TNO V3806. Zeist, Netherlands: TNO (2002).

[20] Berger-Preiss E Koch W Gerling S Kock H Appel KE. Use of biocidal products (insect sprays and electro-vaporizer) in indoor areas--exposure scenarios and exposure Modeling. Int J Hyg Environ Health. (2009) 212:505–18. doi: 10.1016/j.ijheh.2009.02.001 19345645

[21] Delmaar C Meesters J. Modeling consumer exposure to spray products: an evaluation of the Consexpo web and Consexpo Nano models with experimental data. J Expo Sci Environ Epidemiol. (2020) 30:878–87. doi: 10.1038/s41370-020-0239-x, PMID: 32555302

[22] OECD (2021). Evaluation of tools and models for assessing occupational and consumer exposure to manufactured nanomaterials – Part II: Performance testing results of tools/models for occupational exposure. Available at: https://one.oecd.org/document/ENV/CBC/MONO(2021)28/En/pdf

[23] Tongeren MV Lamb J MacCalman L Basinas I Cherrie JW Hesse S. Validation of lower tier exposure tools used for REACH: Comparison of tools estimates with available exposure measurements. Ann Work Expo Health. (2017) 61:921–38. doi: 10.1093/annweh/wxx056 29028246

[24] Jayjock MA Armstrong T Taylor M. The Daubert standard as applied to exposure assessment Modeling using the two-zone (NF/FF) model estimation of indoor air breathing zone concentration as an example. J Occup Environ Hyg. (2011) 8:D114–22. doi: 10.1080/15459624.2011.624387, PMID: 22017382

[25] ECETOC (2022). Systematic review of published studies of Ecetoc TRA worker exposure predictions. Available at: https://www.ecetoc.org/publication/tr-140-systematic-review-of-published-studies-of-ecetoc-tra-worker-exposure-predictions/

[26] Tischer M Meyer J. A new model algorithm for estimating the inhalation exposure resulting from the spraying of (semi)-volatile binary liquid mixtures (SprayEva). Int J Environ Res Public Health. (2022) 19:13182. doi: 10.3390/ijerph192013182, PMID: 36293762 PMC9603233

[27] Koivisto AJ Spinazzè A Verdonck F Borghi F Löndahl J Koponen IK. Assessment of exposure determinants and exposure levels by using stationary concentration measurements and a probabilistic near-field/far-field exposure model. Open Res Europe. (2021) 1:72. doi: 10.12688/openreseurope.13752.1, PMID: 37645135 PMC10446057

[28] Isaacs KK Glen WG Egeghy P Goldsmith M-R Smith L Vallero D. Sheds-Ht: an integrated probabilistic exposure model for prioritizing exposures to chemicals with near-field and dietary sources. Environ Sci Technol. (2014) 48:12750–9. doi: 10.1021/es502513w, PMID: 25222184

[29] Hofstetter E Spencer JW Hiteshew K Coutu M Nealley M. Evaluation of recommended Reach exposure Modeling tools and near-field, far-field model in assessing occupational exposure to toluene from spray paint. Ann Occup Hyg. (2013) 57:210–20. doi: 10.1093/annhyg/mes062, PMID: 23002273

[30] Cherrie JW Maccalman L Fransman W Tielemans E Tischer M Van Tongeren M. Revisiting the effect of room size and general ventilation on the relationship between near- and far-field air concentrations. Ann Occup Hyg. (2011) 55:1006–15. doi: 10.1093/annhyg/mer092, PMID: 22021819

[31] Zhang Y Banerjee S Yang R Lungu C Ramachandran G. Bayesian Modeling of exposure and airflow using two-zone models. Ann Occup Hyg. (2009) 53:409–24. doi: 10.1093/annhyg/mep017, PMID: 19403840 PMC2732913

[32] Nicas M Neuhaus J. Predicting benzene vapor concentrations with a near field/far field model. J Occup Environ Hyg. (2008) 5:599–608. doi: 10.1080/15459620802282375, PMID: 18615292

[33] Cherrie JW Schneider T. Validation of a new method for structured subjective assessment of past concentrations. Ann Occup Hyg. (1999) 43:235–45. doi: 10.1016/S0003-4878(99)00023-X

[34] Cherrie JW. The effect of room size and general ventilation on the relationship between near and far-field concentrations. Appl Occup Environ Hyg. (1999) 14:539–46. doi: 10.1080/104732299302530, PMID: 10462849

[35] Koch W. (2004). Workplace exposure from the use of biocidal products - transformation and extension of a computer based model for the assessment of inhalation and dermal exposure during spray application. Report BAuA, project F2022. Contract No.: Report BAuA project F2022. Available at: https://www.baua.de/DE/Angebote/Publikationen/Berichte/Gd11.html

[36] Commission recommendation (EU) 2022/2510 of 8 December 2022 establishing a European assessment framework for ‘safe and sustainable by design’ chemicals and materials. Available at: https://eur-lex.europa.eu/legal-content/EN/TXT/?uri=CELEX%3A32022H2510

[37] Caldeira C Farcal R Garmendia Aguirre I Mancini L Tosches D Amelio A. Safe and sustainable by design chemicals and materials - framework for the definition of criteria and evaluation procedure for chemicals and materials. Luxembourg: Publications Office of the European Union (2022).

