# Supplementary material for: A generic approach to estimate airborne concentrations of substances released by indoor spray processes using a deterministic 2-box model
Source: Front Public Health. 2024 Feb 9;12:1329096. doi: 10.3389/fpubh.2024.1329096 (PMC10884264; doi:10.3389/fpubh.2024.1329096)
Supplement: Supplementary file 1 [file Data_Sheet_1.pdf]

Supplementary Material

A generic approach to estimate airborne concentrations of substances released by indoor spray processes using a deterministic 2-box model

Stefan Hahn*, Katharina Schwarz, Norman Nowak, Janine Schwarz, Jessica Meyer, Monika Krug, Wolfgang Koch

*** Correspondence:** Corresponding Author: stefan.hahn@item.fraunhofer.de

1 Supplementary Information

1.1 List of symbols

	**Latin symbols**	
$\tilde{c}_{\phi_i}(t, x_{p0})dx_{p0}$	Concentration of active substance in the size range between x_{p0} and $x_{p0} + dx_{p0}$	kg/m³
$\tilde{C}_{\phi_i}(t)$	Time dependent concentration of the (active) substance	kg/m³
$\bar{\tilde{C}}_{\phi_i}(t)$	TWA concentration of the (active) substance	kg/m³
D	Diffusion constant of solvent vapour in air	m²/s
H_s	Settling height	m
k_B	Boltzmann constant	kg m²/(s² K)
$m_0\left(x_{p,0}\right)$	Normalized spray droplet mass distribution density	1/m
$\dot{M}$	Mass flow rate of spray formulation	kg/s
P_{sat}	Vapour pressure of solvent	Pa
S_r	Saturation ratio of solvent vapour	
t_c	Droplet evaporation time	s
T_{air}	Air temperature	K
v_{mo}	Molecular volume	m³
v_s	Settling velocity	m/s
V	Room volume	m³
$x_p(t)$	Droplet diameter at time t	m

$x_{p,0}$	Initial droplet diameter	m
	**Greek symbols**	
α	Evaporation pre-factor, rate of surface shrinkage	m²/s
β	Settling pre-factor	1/m²
ϕ	Mass fraction of the non-volatile substances	
$\hat{\phi}$	Volume fraction of the non-volatile substance	
ϕ_i	Mass fraction of the (active) substance, i	
$\rho_{\phi,i}$	Density of the (active) substance, i	kg/m³
ρ_s	Density of solvent	kg/m³
Γ	Ventilation rate	1/s
η_{air}	Viscosity of air	kg/(m s)

1.2 Analytical spray model

The analytical spray model in order to calculate the correction factors to the generic approach takes into account the normalized mass distribution density of the initially generated droplets, $m_0(x_{p,0})$ as function of the initial droplet diameter, $x_{p,0}$, the time dependent droplet shrinkage due to solvent evaporation and the losses caused by settling on the floor.

Droplet shrinkage kinetics is given by Friedlander (2000)[1]:

$$\frac{dx_p}{dt} = -\frac{\alpha}{x_p} \text{ with } \boldsymbol{\alpha} = \frac{4\,\mathrm{Dv_{mo}P_{sat}}}{\mathrm{k_B T_{air}}}(\mathbf{1} - \mathbf{S_r}) \qquad \textit{Eq. S1}$$

ending at $x_p = x_{p,0}\hat{\phi}^{1/3}$ with $\hat{\phi} = \phi\ \rho_s/\rho_\phi$ and $\rho_\phi^{-1} = \frac{1}{\phi}\sum_{i=1}^{N}\rho_{\phi i}^{-1}\phi_i$. Integration yields:

$$\boldsymbol{x_p(t) = \sqrt{x_{p,0}^2\left(1 - \hat{\phi}^{2/3}\right) - 2\alpha t}.} \qquad \textit{Eq. S2}$$

[1] Friedlander SK. *Smoke, Dust and Haze, Fundamentals of Aerosol Dynamics, 2nd Ed.* New York, USA: Oxford University Press (2000).

The corresponding evaporation time of the droplet is

$$\boldsymbol{t_c = \frac{1}{2\alpha} x_{p,0}^2 \left(1 - \hat{\phi}^{2/3}\right)} \qquad \textit{Eq. S3}$$

Here it is assumed that the total solvent mass fed into the room is small and the temporal change in saturation ratio, S_r, is neglectable during spraying. The time dependent settling velocity is calculated from (Hinds, (1998)[2]):

$$\boldsymbol{v_s = \beta x_p^2(t),} \quad \boldsymbol{\beta \approx \beta_1 = \frac{g\rho_s}{18\,\eta_{air}}} \text{ for } \mathbf{t < t_c} \text{ and } \boldsymbol{\beta = \beta_2 = \frac{g\rho_\phi}{18\,\eta_{air}}} \text{ for } \mathbf{t \geq t_c}. \qquad \textit{Eq. S4}$$

Here it is assumed that during the entire evaporation process the droplet density is the same as the solvent density, ρ_s, and equals the density of the non-volatile substance, ρ_ϕ.

The concentration, $c_{\phi_i}(x_{p,0}, t)$ of the non-volatile substance stemming from the droplets of the initial size range between $x_{p,0}$ and $x_{p,0} + dx_{p,0}$ is calculated from the mass balance between the source strength $\dot{M}\phi_i m_0(x_{p,0})/V$ (where V can be the room volume or the personal volume) and the sinks caused by the time dependent settling rate, $\frac{\beta}{H_s}\left(x_{p,0}^2 - 2\alpha t\right)$, (Eq. S4) and the air exchange rate, Γ:

$$\boldsymbol{\frac{d\tilde{c}_{\phi_i}(x_{p,0},t)}{dt} = -\left[\frac{\beta}{H_s}\left(x_{p,0}^2 - 2\alpha t\right) + \Gamma\right] \tilde{c}_{\phi_i}(x_{p,0}, t) + \dot{M}(t)\phi_i m_0(x_{p,0})/V} \qquad \textit{Eq. S5}$$

where $H_s$is the setting height, in the first instance it may be assumed to be the room height. The mass of substance i in each individual droplet of initial diameter, $x_{p,0}$is constant, irrespective of the droplet's state of evaporation. The solution of Eq.S5 is obtained by separation of variables. Integration over the initial droplet diameter, $x_{p,0}$ yields the temporal concentration pattern of the non-volatile substance

$$\boldsymbol{\tilde{C}_{\phi_i}(t) = \phi_i/V \int_0^\infty m_0(x_{p,0}) \int_0^t \dot{M}(t')\, g(x_{p,0}, t - t') dt' dx_{p,0}} \qquad \textit{Eq. S6}$$

with

$$\boldsymbol{g(x_{p,0}, t) = e^{-\frac{\beta}{H_s}t(x_{p,0}^2 - \alpha t)} e^{-\Gamma t}} \qquad \text{for } \boldsymbol{t < t_c} \qquad \textit{Eq. S7}$$

and

[2] Hinds WC. *Aerosol Technology; Properties, Behavior, and Measuremnt of Airborne Particles, 2nd Ed.* New York, USA: John Wiley & Sons, INC. (1999).

$$g(x_{p,0}, t) = e^{-\frac{\beta_1}{H_s} t_c (x_{p,0}^2 - \alpha t_c)} e^{-\frac{\beta_2}{H_s} x_{p,0}^2 \hat{\phi}^{2/3} (t - t_c)} e^{-\Gamma t} \qquad \text{for } \mathbf{t} \geq \mathbf{t_c} \qquad \textit{Eq. S8}$$

The TWA concentrations of the analytical model applied to the near field and far field volume, $\bar{\bar{C}}_{\phi_i,nf}$ and $\bar{\bar{C}}_{\phi_i,ff}$ are obtained by time integration of Eq. S6.

2 Supplementary Figures and Tables

Additional information is presented in a table which is available as an excel file (Supplemental material – scenarios.xlsx). This table contains information and the coding on the scenarios used for evaluation of the models. For details please refer to the original publications.
